# Adoption of minimally invasive mitral valve surgery in the National Health Service: a blend of science, psychology and human factors

**DOI:** 10.1093/icvts/ivad028

**Published:** 2023-03-10

**Authors:** Megan Joffe, Steven Hunter, Roberto Casula, Inderpaul Birdi, Ranjit Deshpande, Toufan Bharami, Paul Modi, Ishtiaq Ahmed, Hunaid Vohra, Narain Moorjani, Joseph Zacharias

**Affiliations:** Edgcumbe Consulting, Bristol, UK; Department of Cardiothoracic Surgery, Sheffield Teaching Hospitals, Sheffield, UK; Department of Cardiothoracic Surgery, St Mary’s Hospital, London, UK; Department of Cardiothoracic Surgery, Essex Heart Centre, Basildon, UK; Department of Cardiothoracic Surgery, Kings College Hospital, London, UK; Department of Cardiothoracic Surgery, Royal Brompton and Harefield Hospitals, London, UK; Department of Cardiothoracic Surgery, Liverpool Heart and Chest hospital, Liverpool, UK; Department of Cardiothoracic Surgery, Royal Sussex County Hospital, Brighton, UK; Department of Cardiothoracic Surgery, Bristol Royal Infirmary, Bristol, UK; Department of Cardiothoracic Surgery, Royal Papworth Hospital, Cambridge, UK; Department of Cardiothoracic Surgery, Lancashire Cardiac Centre, Blackpool, UK

**Keywords:** Minimally invasive Mitral valve surgery adoption, Innovation, Psychology in surgical innovation, Qualitative research

## BACKGROUND

Minimally invasive mitral valve surgery (MIS) was introduced to the UK National Health Service (NHS) 24 years ago following interest created by American surgeons in the mid-1990s. They first experimented with dogs using long instruments to reach the heart through the ribs, rather than the breastbone, before introducing it to humans [1]. Since then, techniques have developed rapidly. Benefits of MIS for patients include less pain, faster recovery and smaller scars. The advantages for the hospital include shorter length of stay [2], and benefits to patients increase the appeal of the service, and in time, the realization of greater income, which may be an incentive in both privatized and nationalized systems. In the UK, many units implemented MIS techniques, but by early 2000, all had reverted to traditional sternotomy surgery, despite MIS gaining traction in Europe [3] and the USA [4].

A small number of UK cardiac surgeons, keen to adopt these techniques safely, honed their nascent interest in these procedures through independent training and mentoring in Europe. They returned to their units to reintroduce MIS in what was experienced as an unwelcoming, at times, hostile climate. Today, there are over 10 units undertaking cardiac MIS, but MIS remains a minority interest. Informal figures indicate that MIS constitutes ∼8% of mitral valve cardiac surgery in the UK while in Germany, with a population only slightly larger than the UK, it represents 55%.

Surgical innovation typically struggles to report evidence along traditional lines, such as randomized control trials, and the surgeons struggled in the early stages of the surgical development process, according to the IDEAL framework [5], to produce such evidence. Support from professional bodies and the wider surgical community continues to present a barrier to attracting junior surgeons to the sub-specialty, and to making MIS more widely available to patients despite individual surgeon experiences being positive and patients reporting high satisfaction. Adoption of MIS is well documented in other surgical specialties. Why then has it been a struggle for MIS cardiac surgery to gain traction and become as popular a procedure in the UK as it has in Europe or the USA?

What makes these early adopters’ experience unique is that this learning was achieved in cardiac surgery with its low margins for error on a background of intense public scrutiny. A small number reported conditional support from a few colleagues and their organizations. The majority reported active resistance in the form of public criticism of their ethics and motives; suspicion about outcomes in the absence of randomized control trials; lack of funding for necessary equipment; resistance to requests to train and form stable operating teams; and lack of curriculum support for training junior surgeons. Although the surgeons did not perceive the lack of support and criticism as personal, these were significant hurdles. Scepticism also played a role, as it does with most innovation. Within this context, introducing MIS mitral surgery has proven a bumpy ride, with some distance yet to go, proving that introducing a new, innovative technology into complex organizations is more difficult than anticipated.

## METHODS

Prompted by one of these surgeons, a group of 10 ‘early adopter’ surgeons, members of the British and Irish Society for Minimally Invasive Cardiac Surgery (BISMICS), who started an MIS programme between 2006 and 2016, were interviewed individually or in groups of 2 and 3 to share their early experiences. Interviews were virtual and of 60–90 min duration. Their services currently cover the length of England and between them they provide potential access to MIS cardiac surgery to nearly 20 million people.

The retelling of their experiences was prompted through open questions and listening to each other’s narrative, which triggered thoughts about similarities and differences in individual experiences. An analysis was conducted by the first and last authors, broadly based on the principles of thematic analysis [6], and was validated by the larger group.

## RESULTS

The results are reported as themes under the following 5 headings.

### The importance of context

#### Systemic risk aversion

Several interviewees intimated that the NHS was always going to be cautious in accepting MIS and that, in practice, surgical innovation is not easy even with collegial and managerial support especially given UK cardiac surgery’s unfortunate history resulting in heightened caution and even mistrust of surgical motivation and innovation. The Bristol Inquiry (2001) reporting on the tragic outcomes in paediatric cardiac surgery (1991–1995) in Bristol, together with other tragedies involving individual surgeons with poor patient outcomes, attracted media interest and threatened surgical endeavour that was not fully evidence based.

National scrutiny of cardiac surgeons was introduced in 2005 with reporting systems showing named surgeon outcomes. While this was intended to provide public reassurance, it received a mixed reception with national bodies championing the initiative [7] and others more fearful. It had the unintended consequence [8] of dampening surgical motivation to innovate or to take on higher-risk patients, even though the data were risk adjusted.

Making sense of these contextual issues seems to have led to a narrowing in the field of attention and the development of a shared mindset that innovation, with its associated risk in cardiac surgery, was not worth the personal or institutional risk. The priority became to reduce risk and perform, i.e. deliver outcomes in the traditionally accepted mode of open surgery rather than innovate. The personal risk associated with pursuing one’s interest in this context was high with strong potential for being either a hero or a villain, the easier option being to take ‘the middle road where you can be assured of a decent life and you won’t appear on the front pages of the tabloids’.

#### Lack of financial support

Funding for innovative cardiac surgery faces challenges not least because of competition from industry-supported transcatheter procedures. The suggestion that cardiac surgery was being cannibalized (i.e. competed against) and might soon be redundant also made persuading others of its value difficult. Given the increasing demand from patients and the vast need for treatment in the developed world, MIS cardiac surgery does have a bright future if innovation is adopted widely [9].

### The organizational context

Whereas MIS is seen by those offering it, especially in the USA and Europe, as an opportunity to offer advanced patient care, attract patients to your hospital and boost organizational reputation and income, in the NHS, interviewees suggested that MIS is perceived as a problem.

The political cycle with changing ministers and NHS strategies, coupled with the rapid turnaround of hospital Chief Executives, and shifting commissioner demands conspire to provide an unstable climate for innovation. The managerial approach, focusing on targets and productivity, also proved a barrier. MIS typically takes longer than open access surgery, thereby occupying valuable theatre space. It is not readily standardized making it difficult to train others quickly or for less-expensive staff to perform it. No tariff changes were made, and although some instrumentation was reusable, and manufacturers were helpful, considerable cost was added to each case. MIS also required anaesthetists and theatre staff competent and confident to support MIS surgical needs and this had staffing implications. From a financial and productivity perspective, the organizations were not incentivized to support MIS.

Some interviewees suggested that certain hospitals believed themselves to be so successful that there was no need to foster innovation; others ‘don’t think big’ or strategically in terms of extending their services. The challenge of attracting managerial support was exacerbated because it is far easier to champion a widely supported programme than one in which only a couple of people show interest. Thus, the organizational mindset proved problematic for some.

Surgeons referred to the importance of ‘buy-in’ from their colleagues and collaboration with management. Only 1 interviewee joined a custom-built unit; 3 were specifically appointed to lead MIS programmes in larger hospitals, thus being in the good starting position of having organizational legitimacy. Where the surgeon was a lone voice, challenges were extremely difficult.

Individuals had to chart their own course, make a business case requiring collegial endorsement, and deft negotiation for which surgeons do not typically have training, skills or patience. Significantly, few had ‘political clout’ at that early stage in their careers to persuade the senior establishment (managerial and surgical) of their cause. Only 2 surgeons had a supportive senior colleague, providing an extra voice and champion in political and managerial discussions and acting as a ‘shield’ leaving the mechanics to the surgeon most interested in MIS.

One surgeon made the point that managers do not see patients in clinic and so cannot readily appreciate the benefits of the surgery seeing it as a costly exercise associated with high risk and disruption. To influence senior management and garner support, another said that he deliberately invited the most senior managers to work with him on the business case, so sharing the organizational consequences. Another was buoyed by the feeling that he had the support of the CEO. A fourth said that he initially resisted productivity pressure to perform >1 case a day so that he and the team could reflect and learn from each case. A fifth reported having to resist pressure from an anaesthetist to work more quickly to finish in time. Through the deliberate choice of senior management, and through managing the pace, these examples underline the importance of finding an organizational advocate and gaining the confidence of other professionals by focusing on governance and reflection and not giving in to peer pressure.

### Collegial support

Collegial support was forthcoming when these surgeons, as young trainees, contacted more established surgeons in the MIS field who were uniformly supportive. The use of contacts through the ‘old boys’ network’ worked for others. Nonetheless, these supporters were often not part of their employing organization.

Where surgeons were specifically employed to an organization sponsoring an MIS programme collegial support was forthcoming, for others when it was offered, it was greatly valued. Most interviewees recognized that they were somewhat alone in their efforts to introduce the initiative to their hospital, recognizing ‘it was never going to be easy’.

The challenge, for some, from working surgical colleagues took the form of implied and explicit lack of support and even threat. This was summed up by 1 interviewee’s comment that, ‘implied threat is voiced when you are asked if you can do the surgery, and then explicit threat can be assumed when you prove you can because you are then viewed as competition’. Only 1 interviewee offered that a senior colleague had said to him ‘let me know how you are getting on and if you need help’.

More insidious undermining was evident in the example of a consultant colleague writing to a senior medical colleague suggesting that they scrutinize the MIS surgeon’s work. Challenges also came from supporting consultants (e.g. intensivists) about the length of time operations were taking. The interviewees commented that these consultants do not see the patient after the procedure when the patient is well again, and perhaps like all those unfamiliar with the positive outcomes, view the process with cynicism.

Comments, reported by this group, make it clear that some surgical colleagues felt threatened by this new technique and those who practise it. While not uncommon in those who feel less willing to engage with innovation, it would be naive to overlook more subtle psychological pressures such as professional jealousy and interpersonal rivalry. These are also likely to have affected the willingness of some to offer collegial support for the few willing to risk their careers for the sake of innovation and patient-centred care despite the inevitable challenges that their motivation might be more about personal glory and improved income.

The MIS surgeons seemed willing to face the risk of collegial opprobrium, perhaps being labelled a ‘black sheep’ for deviating from the norm and being side lined by the in-group [10], i.e. other cardiac surgeons, the group they are most likely to identify with and risk being ‘isolated’ from, that is, the establishment. From an ethnicity perspective, only one of the surgeons from this early cohort is White British, 4 others were born in the UK to Asian parents and the remaining 5 were immigrants. Thus, they could be viewed as a group that was not typically part of the establishment.

### The theatre team

The surgeons grappled with whether the procedures should be branded as ‘special’ needing a specially trained team or whether MIS should be simply another procedure and cited the former as one of the biggest challenges few managed to achieve in the long term. Most achieved a stable team, at the very least for initial training, but they were required eventually to work in what was a described by one as a ‘Toyota type model’ of working [11]. Customized units had fully trained regular teams; in others, surgeons had to adapt to working with a wide range of theatre staff and work with teams where there was no previous collective learning [12]. Theatre management was unconvinced of the need for stable teams when they were promoting the idea of interchangeable and multi-skilled theatre staff in the interests of productivity.

Given the need to focus on a very narrow field of surgical vision in these MIS cases, situational awareness is the responsibility of the team. Trust, achieved through shared experience of working together, is essential to the development of interdependence. All surgeons emphasized the importance of actively promoting psychological safety, i.e. a shared belief held by members of a team that the team is safe for interpersonal risk taking [12], so that any team member would feel confident to ask a question or voice a concern.

The cost of even the smallest mistake, while extremely high, might be mitigated by reverting to open surgery but the associated criticism would not serve the MIS cause. Whereas similarly serious mistakes might occur in open surgery, a mistake in MIS attracted disproportionate criticism and led to comments, which confirmed the naysayers’ view that mistakes were due to the minimal access and that MIS was dangerous. The effect of confirmation bias [13] proved a strong barrier. Having a second surgeon as another pair of eyes was paramount, and reassuring for the team, but, uncommon 15 years ago. Having the theatre team trained and similarly attuned to the procedure and its risks acted as a safety net.

All interviewees started with a team (anaesthetist, perfusionist and scrub nurse) who accompanied them to be trained by an experienced team. They spoke of instilling pride in the team and making each professional feel ownership of their part in the surgery. Their approach reflected the four-step process of enrolment, preparation, trials and reflection [13] for implementing innovation but, as is evident, was not smooth.

Choosing the right team proved a challenge with 1 surgeon saying that the senior staff wanted to be involved but that he chose those he trusted most and who ‘wouldn’t surprise me’ underlining the importance of trust and suggesting competence trumps experience. Another explained that his choice of team led to accusations and allegations of racism, sexism, perfectionism and elitism. He said that jealousy increased as the team became successful.

### The surgeon

Their determination in the face of the contextual challenges and lack of obvious support raises the question whether they possess attributes which differentiate them from others. The following 5 sets of personal characteristics were evident in the way the interviewees described themselves.

#### Resilience and tolerance for ambiguity

All surgeons reported awareness of the possible risk to their professional, and consequently personal lives, of pursuing and mastering MIS. Two comments typified the sentiment: ‘I gave my whole soul to MIS’ and ‘It’s taken a lot of blood, sweat and tears’*.* It was noted that 5 of the 10 surgeons interviewed were immigrants to the UK and had already experienced major disruption to their lives before embarking on the MIS journey, suggesting a degree of resilience that might be greater than average. They all talked about coping with pressure and stress, not only related to the operation, but that contributed by the context. They agreed that they were ‘comfortable being uncomfortable’.

#### Horizon scanning

They felt early in their surgical career that they could not envisage doing the same thing for the rest of their career. This desire for challenge and curiosity combined with what might be horizon scanning, i.e. checking and anticipating where surgical innovation might take the profession, combined to drive them.

#### Patient centered

Their absolute belief that MIS was better for patients than open heart surgery intensified their determination. There was no definitive science to support the adoption of these procedures and it fell on them to both innovate and create the science to support their patient-centred beliefs.

#### Planning and preparation

The interviewees talked about the need for planning, preparation and focused concentration before and during the operation. One said that his planning and preparation took 3 years before he performed his first procedure.

#### Confidence and self-awareness

Another commonality was strong self-belief and confidence. All interviewees were aware that they might be perceived as arrogant rather than simply confident. Underneath this confidence was persistent self-questioning. They spoke about the need for constant reflection and continuous learning exemplified by the following comment: ‘When I look back on my proficiency 3 years ago, I can’t believe I thought that was good’.

#### Mental agility and reflection

Manual dexterity, spatial ability and perceptual motor skills notwithstanding, the need to make quick decisions and develop a plan B was emphasized as a characteristic of all surgeons but without the help of a colleague, the cognitive flexibility in navigating the complexity of MIS was different to what is typically required. Even experienced MIS surgeons do some open surgery and some felt that MIS improved their open surgery.

How these surgeons framed their role and shaped their behaviour is likely to have had a significant impact on their willingness to take the risk and innovate despite the heightened sensitivity and potential condemnation. Dweck and Legget’s notion of performance versus learning orientation is likely relevant and to have driven self-questioning and reflection. They differentiate the former as focused on the goal and outcome and the latter more focused on learning [14]. The message from all surgeons was clear; they needed a successful outcome and an openness to reflective learning.

## CONCLUSIONS

The interviewees’ experience ranged from 2006 to 2016 demonstrating that this specific innovation did not become easier with time. On reflection, these early adopters suggested that the technical challenges were the least of their problems because they were careful to be trained, proctored and mentored and, in fact, were not the original pioneers but were following those who had broken the ground for them. What was more difficult to manage and understand was the lack of collegial, organizational and systemic support. In retrospect, exploring their own psychology, attempting to understand that of their colleagues, and the psychology of the system, might have made their journey easier. Research [15] suggests that the successful surgeon innovator must be savvy in medicine, technology and business, but this study shows that an understanding of psychology and human factors is central too. There was unanimous agreement that the publication of surgeon-specific results did not help the cause of adopting new techniques within teams.

The hope is that lessons learned from this exercise will help the wider adoption of other surgical procedures like endoscopic conduit harvesting and minimally invasive coronary artery surgery and may have relevance for other surgical specialties too.

## Notes

This work was presented at EACTS 2021.
